# Outcome of liver cirrhosis patients requiring prolonged mechanical ventilation

**DOI:** 10.1038/s41598-020-61601-2

**Published:** 2020-03-18

**Authors:** Chih-Cheng Lai, Kuei-Ling Tseng, Chung-Han Ho, Shyh-Ren Chiang, Khee-Siang Chan, Chien-Ming Chao, Shu-Chen Hsing, Kuo-Chen Cheng, Chin-Ming Chen

**Affiliations:** 1 1Department of Internal Medicine, Kaohsiung Veterans General Hospital, Tainan Branch, Tainan, Taiwan; 2 Departments of Internal Medicine, Chi Mei Medical Center, Tainan, Taiwan; 3 Department of Medical Research, Chi Mei Medical Center, Tainan, Taiwan; 4 Department of Intensive Care Medicine, Chi Mei Medical Center, Tainan, Taiwan; 5 Department of Hospital and Health Care Administration, Chia Nan University of Pharmacy & Science, Tainan, Taiwan; 6 Department of Safety Health and Environmental Engineering, Chung Hwa University of Medical Technology, Tainan, Taiwan; 7 Department of Intensive Care Medicine, Chi Mei Medical Center, Liouying, Taiwan

**Keywords:** Liver cirrhosis, Disease-free survival

## Abstract

Acute respiratory failure requiring mechanical ventilation is a major indicator of intensive care unit (ICU) admissions in cirrhotic patients and is an independent risk factor for ICU mortality. This retrospective study aimed to investigate the outcome and mortality risk factors in patients with liver cirrhosis (LC) who required prolonged mechanical ventilation (PMV) between 2006 and 2013 from two databases: Taiwan’s National Health Insurance Research Database (NHIRD) and a hospital database. The hospital database yielded 58 LC patients (mean age: 65.3 years; men: 65.5%). The in-hospital mortality was significantly higher than in patients without LC. Based on the NHIRD database of PMV cases, patients were age-gender matched in a ratio of 1:2 for patients with and without LC. Model for End-Stage Liver Disease (MELD) score was calculated. The mortality was higher in patients with LC (19.5%) than those without LC (18.12%), though not statistically significant (p = 0.0622). Based on the hospital database, risk factor analysis revealed that patients who died had significant higher MELD score than the survivors (18.9 vs 13.7, p = 0.036) and patients with MELD score of >23 had higher risk of mortality than patients with MELD score of ≤23 (adjusted OR:9.26, 95% CI: 1.96–43.8). In conclusion, the in-hospital mortality of patients with high MELD scores who required PMV was high. MELD scores may be useful predictors of mortality in these patients.

## Introduction

The health burden of liver cirrhosis (LC) has significantly increased worldwide^1^; it resulted in one million mortalities in 2010 according to the Global Burden of Disease Study 2010 and is estimated to cost 31 million Disability Adjusted Life Years (DALYs)^2,3^. In Taiwan, liver cirrhosis mortalities jumped from 3723 in the 1980s to 6243 in 2010s^1^. In patients with previously compensated liver cirrhosis, several conditions such as variceal bleeding, ascites, hepato-renal syndrome, hepatic encephalopathy, hepato-pulmonary syndrome, spontaneous bacterial peritonitis, and severe sepsis may result in unstable clinical conditions that require admission to an intensive care unit (ICU). Once a patient with LC is admitted to the ICU, the mortality rate can range from 34% to 69%^4^.

Acute respiratory failure that requires mechanical ventilation (MV) is a major indication for the requirement of ICU admission for cirrhotic patients and is an independent risk factor of ICU mortality^5–7^. Although the mortality rate of critically ill patients with LC who need MV is >50%^8–10^, critically ill patients might survive the acute stage in the ICU and enter the difficult stage of being weaned off MV and therefore, may require prolonged mechanical ventilation (PMV). Such patients require a lot of healthcare resources and medical expenses; however, their prognoses are unclear and difficult to assess. Being able to predict whether an LC patient will need PMV and be able to predict their outcomes would allow for healthy mutual communication between physicians, patients, and their family to arrive at a consensus about their post-PMV care plans. Once the outcome can be fully understood and the prognosis can be accurately predicted, physicians can help patients and their families make the best choice regarding the use of PMV. However, investigations focusing on the prognoses and outcomes of patients with LC requiring PMV are scarce. Only one recent study has reported the median survival (0.19 years) and life expectancy (3.59 years) of 1478 LC patients requiring PMV^11^. Thus, this study aims to investigate the outcome of the cirrhotic patients requiring PMV using two databases – The national health insurance research database (NHIRD) and a hospital-based database. Further, we used detailed information obtained from the hospital-based database to identify risk factors associated with patients’ mortality.

## Methods

### Patients and study setting

This retrospective study was conducted at Chi Mei Hospital, a 1288 bed tertiary medical center in Tainan City, Taiwan. The hospital has 110 adult intensive care unit (ICU) beds and 16 respiratory care center (RCC) beds. The RCC is prepared to care for difficult-to-wean ICU patients. If the critical patients show improvement including hemodynamic stability without vasoactive agents but required oxygenation, had no acute liver or kidney failure, and appeared not to require any more surgical intervention in the near feature, their in-charge ICU physician considers them eligible for transfer from the RCC.

All patients with LC transferred to the RCC between 2006 and 2013 were identified from the NHIRD database; patients who required MV for more than three weeks were included in this study. To assess the effects of LC on the outcome of PMV patients, we used a 1:2 ratio of age-sex matched cohort of LC^[positive (+)]^ and LC^[negative (–)]^ patients. The retrospective data were collected on a routine basis and the analysis was carried out retrospectively. Therefore, no informed consent was required, and it was specifically waived off by our Institutional Review Board (IRB). Ethical approval was obtained from Chi Mei Medical Center (IRB 10411-E01). All methods were performed in accordance with the relevant guidelines and regulations. To effectively use MV and reduce the heavy utilization of the ICU, the Taiwan Bureau of National Health Insurance (BNHI) implemented an integrated prospective payment program (IPP) for patients in Taiwan who required PMV in 2000^11^. Based on this policy, PMV patients were required to be transferred to an RCC after an ICU stay of 21 days and to a respiratory care ward after an RCC stay of 42 days^11–13^. The NHIRD database provides detailed information of all insured inpatients and outpatients using the International Classification of Diseases, Ninth Revision, Clinical Modification (ICD-9-CM) code. Encrypted personal identification was used to protect the identities of the individuals in the database.

### Parameters assessed

The medical records of all the LC patients with PMV were retrospectively reviewed and the following information was collected: age, gender, category of previous ICU stay, length of ICU and RCC stays, diagnosis of RCC admission, duration of MV use in ICU and RCC, Acute Physical and Chronic Healthy Evaluation II (APACHE II) score on ICU and RCC admission, serum albumin level, blood urea nitrogen (BUN) level, electrolytes, and serum creatinine level. The severity of LC was determined using the Model for End-Stage Liver Disease (MELD) score based on the result of blood tests upon RCC admission. In-hospital mortality (death due to any cause) was our primary outcome measure.

## Statistical Analysis

Continuous variables are expressed as mean ± standard deviation (SD). The significance of the difference between categorical variables and continuous variables between groups was tested using the χ^2^ test or one-way analysis of variance, respectively. A multivariable logistic regression model was constructed from baseline characteristics and clinical variables with p-values <0.05 as candidates. For determining the final prediction model, the stepwise model-selection procedure (in which all candidate variables were inserted until non-effects entered or the effects were removed by backward elimination) was used to examine the association between predictive variables and the mortality rate using odds ratios (OR) with 95% confidence intervals (95% CI). In addition, we used MELD score of 23 as a cut-off value for further analysis based on a previous study^14^. All statistical analyses were conducted using SPSS 19 for Windows (IBM, Armonk, NY, USA). Significance was set at p < 0.05.

## Results

### Outcome of LC patients with PMV

Fifty-eight LC^[+]^ PMV patients (mean age: 65.3 years; men, 65.5%) were identified from the hospital database. Forty-five patients with LC requiring PMV were transferred from the medical ICU to the RCC unit. Pneumonia (50%) and neuromuscular disease (32.8%) were the most frequent causes of RCC admission. The mean length of RCC stay was 16.8 days and the in-hospital mortality was 29.3% (Table 1). The causes of death included sepsis (n = 10), variceal bleeding (n = 3), intracerebral hemorrhage (n = 2), hepatorenal syndrome (n = 1), and hepatic encephalopathy (n = 1). For sepsis-associated mortality, pneumonia was the most common type of infection (n = 5), followed by peritonitis (n = 3), and central venous catheter-related infection (n = 2). Additionally, in two patients, the endotracheal tube was withdrawn for hospice care.

**Table 1:** Clinical variables characteristics of all patients.

Variable	LC (*n* = 58)	non-LC (*n* = 174)	P value
**Age (years), mean** ± **SD**	65.3 ± 11.7	65.3 ± 11.6	1.000
**Gender, n(%)**			
Male	38(65.5)	114(65.5)	1.000
Female	20(34.5)	60(34.5)	
**Category of transferred ICU, n(%)**			0.008
Surgery	13(22.4)	73(42.0)	
Medicine	45(77.6)	101(58.0)	
**Length of ICU stay, mean** ± **SD**	20.08 ± 9.2	17.7 ± 9.1	0.025
**ICU APACHE II scores, mean** ± **SD**	22.1 ± 7.5	20.9 ± 7.5	0.324
**RCC APACHE II scores, mean** ± **SD**	18.8 ± 5.7	18.5 ± 5.6	0.723
**Hemodialysis, n(%)**			0.108
None	44(75.9)	148(85.1)	
HD	14(24.1)	26(14.9)	
**Diagnosis of RCC admission, n(%)**			0.032
Pneumonia	29(50.0)	64(36.8)	0.075
Decompensated heart disease	2(3.4)	17(9.8)	0.128
Neuromuscular disease	19(32.8)	82(47.1)	0.056
Infection other than pneumonia	4(6.9)	6(3.4)	0.263
Decompensated gastrointestinal disease	4(6.9)	3(1.7)	0.046
Others	0(0)	2(1.1)	1.000
**Laboratory examinations at RCC admission, mean** ± **SD**			
Blood urea nitrogen, mg/dl	47.8 ± 30.9	42.2 ± 30.0	0.220
Creatinine, mg/dl	1.9 ± 1.8	1.8 ± 1.8	0.848
Albumin, g/dl	2.6 ± 0.6	2.7 ± 0.5	0.322
Hemoglobin, g/dl	9.8 ± 1.3	10.0 ± 1.6	0.469
Phosphate, mg/dl	3.2 ± 1.5	3.6 ± 1.0	0.084
**Outcomes**			
**Length of RCC stay, mean** ± **SD**	16.8 ± 10.7	16.7 ± 9.4	0.913
**In-hospital mortality, n(%)**	17(29.3)	37(21.3)	0.209

ICU, intensive care unit; RCC, respiratory care center; LC, liver cirrhosis.

On comparing this population with the age-sex matched cohort from the NHIRD database, we observed that LC^[+]^patients requiring PMV were more likely to be transferred to the RCC unit from medical ICU, had a longer length of ICU stay, and had decompensated gastrointestinal diseases causing RCC admission, than LC^[–]^ patients; all these were statistically significant (p < 0.05). There were no significant differences between patients with and without LC; these two cohorts had similar outcomes in terms of length of RCC stay and in-hospital mortality.

We identified 6059 LC^[+]^ patients in the NHIRD and 23,806 LC^[–]^ patients who had undergone PMV. Patients with LC in the NHIRD were younger, had more males, and had significantly more HBV and HCV infections (all p < 0.05). The in-hospital mortality was higher among LC^[+]^ patients than among LC^[–]^ patients (p = 0.0049). However, after age and gender matching age and gender, it was seen that patients with LC had higher mortality than those without LC (19.5% vs 18.12%, p = 0.0622) (Table 2).

**Table 2:** Demographic characteristics of prolonged mechanical ventilation patients with and without liver cirrhosis (LC).

	Before Matching			After Matching (1:2)		
	Non-LC (N = 23806)	LC (N = 6059)	p-value	Non-LC (N = 8236)	LC (N = 4118)	p-value
**Age, mean** ± **SD**	69.36 ± 12.46	68.27 ± 12.47	<0.0001	71.28 ± 10.67	71.28 ± 10.67	1.0000
**Gender, n(%)**						
Male	13658 (57.37)	3730 (61.56)	<0.0001	5042(61.22)	2521(61.22)	1.0000
Female	10148 (42.63)	2329 (38.44)		3194(38.78)	1597(38.78)	
**Comorbidity, n(%)**						
hepatitis B virus	198(0.79)	96(1.58)	<0.0001	73(0.89)	55(1.34)	0.0201
hepatitis C virus	109(0.46)	84(1.39)	<0.0001	33(0.40)	53(1.29)	<0.0001
**Death, n(%)**						
Survival	19686 (82.69)	4917 (81.15)	0.0049	6744(81.88)	3315(80.50)	0.0622
Expired	4120 (17.31)	1142 (18.85)		1492(18.12)	803(19.50)	

### Factors predicting in-patient mortality of LC^[+]^ patients requiring PMV

**Figure 1:**
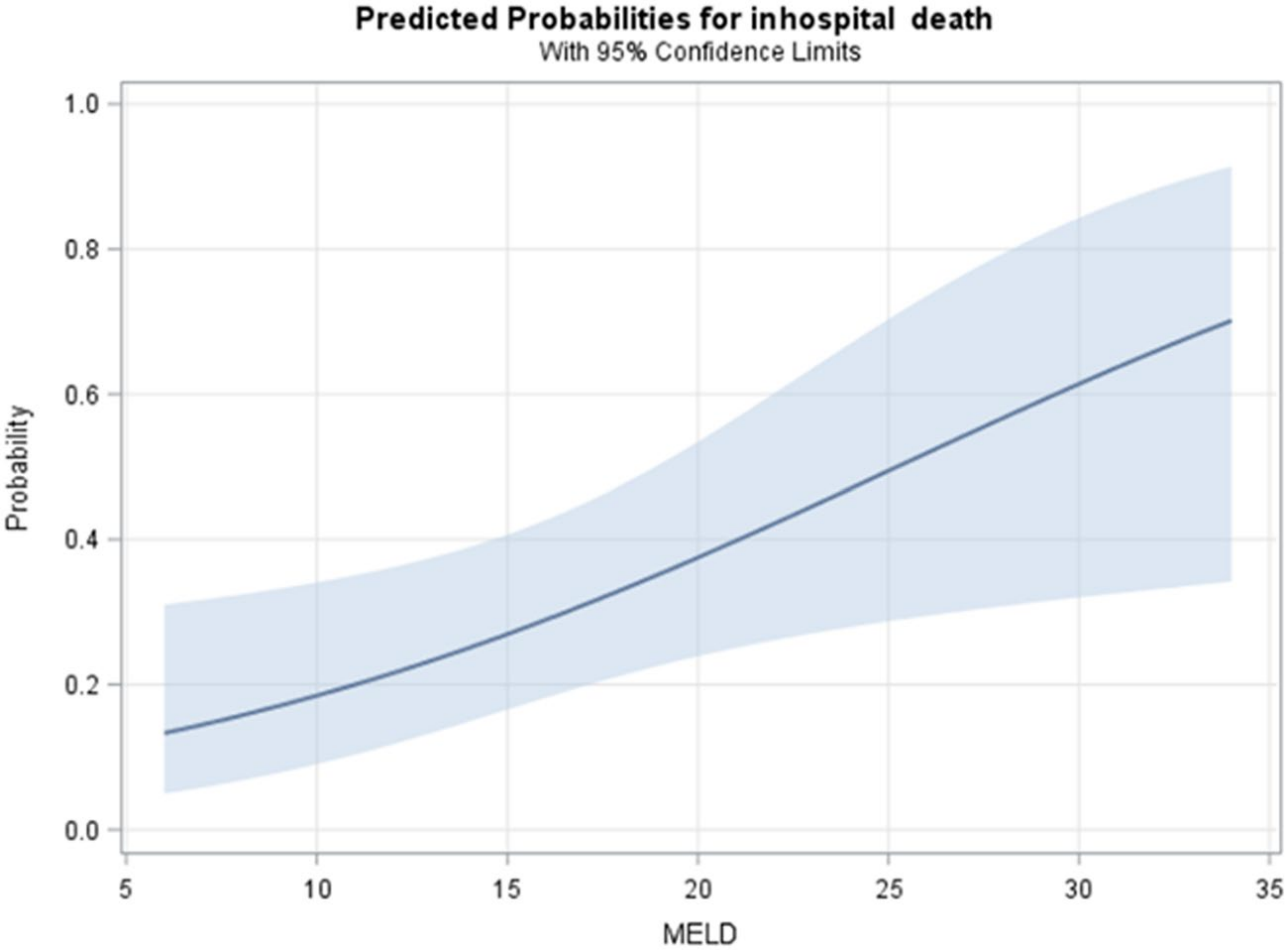
Association between MELD score and mortality.

**Figure 2:**
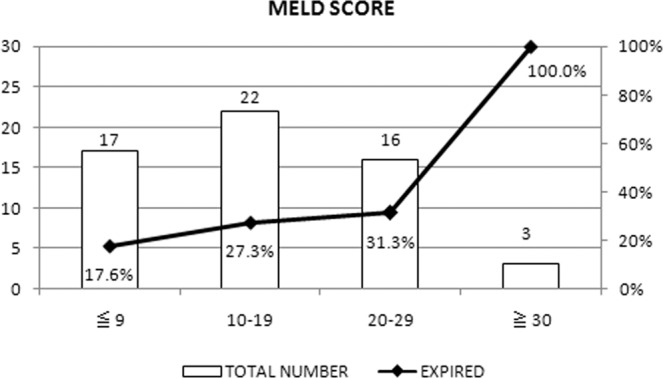
The predicted probabilities for in-hospital deaths using the MELD score.

Among the 58 LC^[+]^ patients requiring PMV, 17 patients suffered from in-hospital mortality. No significant difference was observed between survivors and those who died in terms of age, sex, category of transferred ICU, length of stay in ICU and RCC, APACHE II score upon ICU and RCC admission, diagnosis of RCC admission, and laboratory findings (all p > 0.05). However, we did observe that those who died showed significantly higher MELD scores than the survivors (18.9 vs 13.7, p = 0.036) (Table 3), and the higher MELD scores was associated with a higher probability for mortality (Figs. 1 and 2). As reported in a previous study^14^, we used 23 as the cut-off value of MELD score for further analysis and observed that patients with MELD ≥ 23 had higher risk of mortality than patients with MELD < 23 (adjusted odds ratio [OR]: 9.26, 95% confidence interval [CI]: 1.96–43.8) (Table 4).

**Table 3:** The comparison of clinical outcome of patients with liver cirrhosis.

Variable	Survival cases (n = 41)	Mortality cases (n = 17)	P value
**Age (years), mean** ± **SD**	65.5 ± 12.0	64.9 ± 11.3	0.867
**Sex, n(%)**			
Male	26(63.4%)	12(70.6%)	0.601
Female	15(36.6%)	5(29.4%)	
**Category of transferred ICU, n(%)**			1.000
Surgery	9(22.0%)	4(23.5%)	
Medicine	32(78.0%)	13(76.5%)	
**Hepatitis B virus, n(%)**	5(12.2%)	3(17.6)	0.681
**Hepatitis C virus, n(%)**	10(24.4%)	3(17.6%)	0.736
**Alcoholism, n(%)**	2(4.9%)	1(5.9%)	1.000
**Length of ICU stay, mean** ± **SD**	21.6 ± 10.2	18.8 ± 5.7	0.295
**Length of RCC stay, mean** ± **SD**	17.5 ± 11.0	15.1 ± 9.9	0.425
**ICU APACHE II scores, mean** ± **SD**	21.3 ± 7.7	23.9 ± 6.6	0.250
**RCC APACHE II scores, mean** ± **SD**	18.3 ± 5.8	20.1 ± 5.3	0.281
**Hemodialysis, n(%)**			0.311
None	33(80.5%)	11(64.7%)	
HD	8(19.5%)	6(35.3%)	
**Diagnosis of RCC admission, n(%)**			0.242
Lung infection	20(48.8%)	9(52.9%)	
Decompensated heart disease	2(4.9%)	0(0%)	
Neuromuscular disease	14(34.1%)	5(29.4%)	
Infection other than pneumonia	4(9.8%)	0(0%)	
Decompensated gastrointestinal disease	1(2.4%)	3(17.6%)	
**Laboratory findings, mean** ± **SD**			
Blood urea nitrogen, mg/dl	45.4 ± 33.6	53.6 ± 22.9	0.359
Creatinine, mg/dl	1.6 ± 1.6	2.6 ± 1.9	0.051
Serum sodium, mEq/L	142.7 ± 6.1	142.1 ± 10.7	0.807
Bilirubin Total, mg/dl	2.4 ± 4.4	3.2 ± 6.0	0.543
Albumin, g/dl	2.7 ± 0.5	2.5 ± 0.6	0.371
Hemoglobin, g/dl	9.9 ± 1.3	9.6 ± 1.4	0.391
Phosphate, mg/dl	3.3 ± 1.5	3.0 ± 1.4	0.517
**MELD score, mean** ± **SD**	13.7 ± 6.2	18.9 ± 8.8	0.036

ICU, intensive care unit; RCC, respiratory care center.

**Table 4:** The risk of mortality in LC patients with MELD score.

	No. of death	Crude OR (95% C.I.)	p-value	Adjusted* OR (95% C.I.)	p-value
MELD score					
<23	10(20.83)	1.00(ref)		1.00(ref.)	
≥23	7(70.00)	8.87(1.94–40.59)	0.0049	9.26(1.96–43.80)	0.0050

The odds ratio (OR) was adjusted by age and gender.

## Discussion

Using the two databases– hospital-based and NHIRD-based, this study investigated the outcome of LC patients requiring PMV. Initially, we found that the overall mortality of this population was 29.3% and 19.5% in the hospital-based and NHIRD-based investigations, respectively. Although mortality rates of LC^[+]^ patients were higher than those of LC^[–]^ patients, the differences were not significant. The lack of significant survival differences between the cirrhotic and non-cirrhotic cohort maybe because we included patients even with mild LC in this study. In the hospital-based cohort, 29.3% of the total LC patients had MELD score less than 10 which indicated less severe liver cirrhosis and the mortality of this subgroup being lower than patients with a MELD score greater than 10. In addition, we also observed that patients who died had a higher MELD score than the survivors (18.9 vs 13.7). To resolve this issue, we further analyzed the data to identify the risk factor of mortality among LC patients; we only found one prognostic factor - MELD score which was significantly associated with mortality among this specific population. The usefulness of the MELD score in predicting the outcome of cirrhosis patients in various conditions has been demonstrated in previous studies^14–17^; however, to the best of our knowledge, our study is the first to show that MELD scores can be associated with mortality in cirrhosis patients requiring PMV. Moreover, by using 23 as a cutoff for MELD score based on a previous study^14^, we observed that patients with a MELD score of ≥ 23 had a higher risk of mortality than patients with MELD of <23. In summary, the findings of this study indicate that not all LC patients requiring PMV had significantly higher mortality compared to those without LC; patients with severe LC (with higher MELD scores) showed significant association with higher mortality than those with mild LC (lower MELD scores).

Knowing the prognostic factors and being able to predict the outcome of LC^[+]^ patients requiring PMV is important for hepatologists, patients, and patients’ families to have better communication and to arrive at a consensus about the patient’s care plans. More than 20% of the patients requiring PMV are more likely to be near the end of their life and some of them may prefer palliative care to preserve the quality of life rather than prolonging their life expectancy by receiving life-sustaining therapy including PMV. Our findings can help us to better understand the outcome of LC patients on PMV and provide important information about the prognosis of this specific population. Further, it suggests that patients with severe LC on PMV are associated with poor outcome and palliative care may be recommended for these patients.

Our study has a few limitations. First, we measured only the in-hospital mortality rate. We did not assess the patient outcomes post-discharge; thus, we are unable to provide data on long-term outcomes, e.g., the one-year survival rate. In addition, we did not evaluate patients’ quality of life. Further studies are needed to clarify these issues. Second, the number of patients from the single hospital-based setting was limited; therefore, our findings cannot be generalized to patients in other hospitals or countries. However, our study did use another large-scale NHIRD database through which we enrolled many patients. Findings from both the databases were similar; thus, the results should remain representative of this specific population. Third, LC^[+]^ severity was only available in the hospital-based setting, but not in the NHIRD. In addition, because some subjective assessments of LC severity in Child classification, such as ascites and hepatoencephalopathy were not available in this retrospective study, we used MELD scores which provided an objective assessment of the disease severity. Finally, due to the retrospective nature of this study, some bias may have confounded our analysis.

## Conclusions

The in-hospital death rate of severe LC^[+]^ patients who required PMV was high and MELD scores may be useful indicators for predicting mortality in this population.
